# Metabolomics Reveals the Anti-hepatic Fibrosis Mechanisms of *Pueraria lobata* (Willd.) Ohwi Extract and Potential Metabolites Alterations

**DOI:** 10.7150/ijms.129139

**Published:** 2026-03-17

**Authors:** Yu-Ju Chen, Lo-Yun Chen, Ngoc-Thac Pham, John Louie Dela Vega, Federica Fulgheri, Ching-Chiung Wang, Maria Letizia Manca, Chia-Jung Lee

**Affiliations:** 1 Ph.D. Program in Clinical Drug Development of Herbal Medicine, Taipei Medical University, Taipei, Taiwan.; 2 Department. of Life and Environmental Sciences, University of Cagliari, University Campus, S.P. Monserrato-Sestu Km 0.700, CA, Monserrato, 09042, Italy.; 3 Ph.D. Program in School of Pharmacy, Taipei Medical University, Taipei, Taiwan.; 4 Graduate Institute of Pharmacognosy, Taipei Medical University, Taipei, Taiwan.; 5 School of Pharmacy, Collage of Pharmacy, Taipei Medical University, Taipei, Taiwan.; 6 Traditional Herbal Medicine Research Center, Taipei Medical University Hospital, Taipei, Taiwan.

**Keywords:** Anti-liver fibrosis, *Pueraria lobata* (Willd.) Ohwi, Network pharmacology, Metabolomics

## Abstract

Liver fibrosis is a progressive disorder characterized by excessive extracellular matrix (ECM) accumulation, leading to impaired liver function and potentially cirrhosis. *Pueraria lobata* (Willd.) Ohwi, a traditional medicinal plant, has shown promise for hepatoprotection. In this study, we evaluated the antifibrotic effects of a 50% ethanol extract of *Pueraria lobata* (PUR50E) using integrated network pharmacology, metabolomics, and a CCl₄-induced mouse model of liver fibrosis. Network pharmacology analysis identified key PUR50E-associated targets involved in ECM organization, oxidative stress regulation, and TGF-β-related fibrogenic signaling pathways. PUR50E markedly reduced ECM markers, including α-SMA and fibronectin, and enhanced ECM remodeling through upregulation of MMP-3 and MMP-13. It also activated the Nrf2/HO-1 pathway to alleviate oxidative stress. Metabolomic profiling revealed key alterations, including reduced malate, fumarate, succinate, and isocitrate in the TCA cycle; decreased tryptophan, indole, and N-acetylserotonin with increased melatonin in tryptophan metabolism; and elevated glycine and homoserine in glyoxylate and dicarboxylate metabolism. These findings suggest that PUR50E mitigates liver fibrosis by promoting ECM degradation, enhancing antioxidant defense, and restoring metabolic homeostasis. The identified metabolites may serve as potential biomarkers and therapeutic targets, supporting the development of PUR50E as a promising multi-target natural therapy for liver fibrosis.

## 1. Introduction

Liver fibrosis is the primary pathological process associated with chronic liver injury, which can be triggered by various etiological factors, including persistent viral hepatitis, immune-mediated damage, alcohol consumption, and toxic insults. If left untreated or without effective interventions, liver fibrosis can advance to cirrhosis, hepatocellular carcinoma (HCC), or even lead to death. Identifying promising novel biomarkers and therapeutic targets is crucial for advancing the development of clinical treatments and improving patient outcomes.

During liver fibrosis, growth factors are considered key contributors to the accumulation of ECM. Among them, transforming growth factor-β1 (TGF-β1) is the most prominent growth factor driving ECM deposition. TGF-β1 plays a pivotal role in maintaining the balance between fibrogenesis and fibrosis by activating its downstream Smad signaling pathway. TGF-β/Smad signaling pathway is regarded as a classic pathway in the fibrotic response. When TGF-β1 binds to its receptors, TGF-β type I receptor (TGF-β RI) and type II receptor (TGF-β RII), it activates receptor kinase activity, leading to the phosphorylation of downstream Smad proteins. These phosphorylated Smad proteins form a complex with the co-Smad, Smad 4, which translocates into the nucleus to regulate the expression of fibrosis-related genes such as α-smooth muscle actin (α-SMA), collagen type I (Col-I), fibronectin, and matrix metalloproteinases (MMPs).

MMPs are a group of critical proteolytic enzymes responsible for degrading ECM components and play a central role in maintaining ECM homeostasis in the liver. In the early stages of liver fibrosis, MMP activity decreases, leading to excessive ECM deposition. During fibrosis regression, however, MMPs are reactivated, facilitating ECM degradation and aiding tissue remodeling 1. Therefore, targeting the TGF-β/Smad signaling pathway and regulating MMP activity are critical strategies for treating liver fibrosis. Nuclear factor erythroid 2-related factor 2 (Nrf2) is a key regulator of cellular defense against oxidative stress, moreover, many natural Nrf2 activators can regulate lipid metabolism and oxidative stress of liver cells to alleviate fatty liver disease in mice 2. Oxidative stress during liver fibrosis enhances the activation of TGF-β signaling, further exacerbating ECM accumulation. However, previous studies have demonstrated that activation of the Nrf2/HO-1 pathway exerts significant anti-fibrotic and antioxidant effects. Exploring the potential of multi-target therapies that combine antioxidative, ECM degradation-promoting, and anti-fibrotic approaches could provide new opportunities for the treatment of liver fibrosis.

Currently, the absence of effective clinical interventions and reliable early biomarkers remains a major challenge in the treatment of liver fibrosis. Therefore, exploring natural compounds with multi-target activities has become an attractive strategy. Traditional Chinese medicine (TCM), which is characterized by its holistic regulation of multiple pathways, offers unique advantages in this regard. *Pueraria lobata* (Willd.) Ohwi, also known as Pueraria lobata radix (PUR) or Kudzu root, is a perennial twining vine of the leguminosae family and one of the medicinal and edible homologous plants. According to the Chinese Pharmacopoeia, PUR has properties such as relieving muscle tension and fever, promoting “yang,” alleviating diarrhea, facilitating rash eruption, and producing bodily fluids to quench thirst. It is used to treat conditions such as diarrhea due to spleen deficiency, thirst caused by fever, exogenous fever, severe headache, poor measles eruption, febrile thirst, alcoholism, chest pain, and angina pectoris 1. Modern pharmacological research has found that PUR primarily contains flavonoids, triterpenoids, saponins, polysaccharides, and other chemical components, which exhibit various biological activities 3. These include neuroprotection 4, cardiovascular and cerebrovascular disease prevention 5, anti-diabetic effects 6, liver protection 7, and improvement of osteoporosis 8. In recent years, various experimental studies have shown that Traditional Chinese Medicine (TCM) can mitigate hepatic fibrosis by reducing oxidative stress, inhibiting HSC activation, and decreasing ECM deposition. These effects help suppress fibrosis and connective tissue proliferation, improve liver function, and slow the progression of hepatic fibrosis. Due to its multi-target properties, PUR is expected to be a promising drug for the treatment of liver fibrosis. However, the specific mechanisms by which PUR inhibits liver fibrosis remain unclear.

Network pharmacology, a systems biology approach, offers significant advantages in investigating the multi-component, multi-target characteristics of herbal medicines. It facilitates the prediction of active compounds, potential targets, and associated signaling pathways, providing a comprehensive overview of the therapeutic landscape 9. However, the reliability of network pharmacology heavily depends on the completeness and accuracy of existing databases and literature. If the data are outdated or incomplete, the results may be less reliable. Moreover, network pharmacology primarily provides theoretical predictions of potential targets and mechanisms, necessitating further experimental validation to confirm their actual biological effects.

Meanwhile, metabolomics provides a powerful platform for identifying disease-associated metabolic alterations and discovering potential biomarkers. By capturing global metabolic profiles, it not only helps elucidate drug mechanisms but also enables the identification of early diagnostic indicators and therapeutic targets in liver fibrosis. In contrast to network pharmacology, which predicts molecular interactions at the gene, protein, and pathway levels, metabolomics reflects real physiological changes by directly measuring metabolites in biological samples 10. Integrating both approaches allows theoretical predictions to be validated through their actual metabolic outcomes, forming a complementary framework that enhances the scientific rigor and reliability of mechanistic studies on PUR. This study employs an integrated approach combining network pharmacology, metabolomics, and experimental validation to explore the anti-fibrotic effects of PUR in a CCl_4_-induced mouse model of liver fibrosis. By leveraging the complementary strengths of these methodologies, this research aims to elucidate the molecular mechanisms underlying PUR's hepatoprotective effects and identify potential therapeutic targets, providing a scientific basis for its future clinical application and the development of novel therapeutic strategies.

## 2. Materials and Methods

### 2.1 Preparation of 50% ethanol extract of *Pueraria lobata* (Willd.) Ohwi (PUR50E)

*Pueraria lobata* (Willd.) Ohwi (PUR) was obtained from Herbiotek Co., Ltd and authenticated by the Brion Research Institute of Taiwan. The PUR sample was ground and sieved through a 20-mesh screen. The sample was extracted with 10 times its weight in 50% ethanol at 85 °C by reflux for 2 hours. The extract was collected, and an additional 10 times volume of 50% ethanol was added for a second reflux extraction for 2 hours. The extracts were combined, filtered through a Büchner funnel, and then concentrated using a rotary evaporator under reduced pressure for further use. The concentrated sample is called PUR50E.

### 2.2 HPLC-QTOF-MS/MS analysis

A SHIMADZU LCMS-9030 system (SHIMADZU Corporation, Kyoto, Japan) was employed to collect MS2 data. Prior to MS analysis, chromatographic separation was conducted using a SHIMADZU Shim-pack GIST C18 column (2 μm, 2.1 mm × 100 mm). The mobile phase consisted of MeCN (A, with 0.1% formic acid) and water (W, with 0.1% formic acid), following this gradient program: 0-35 min, 5-35% A; 40 min, 100% A; 40.1-45 min, 5% A. The column temperature was maintained at 40 °C, and the flow rate was set at 0.4 mL/min. The sample was prepared by diluting 1.0 mg of the extract in 1 mL of methanol (200 ppm) and then filtered through a 0.22 μm membrane. Automatic sample injection was carried out with a 5 μL injection volume. For MS settings, the interface voltage was 4.0 kV in positive-ion mode and -3.0 kV in negative-ion mode. The nebulizing gas flow rate was maintained at 3.0 L/min, and MS1 and MS2 spectra were acquired across the *m/z* range of 100-1800. MS2 data acquisition used an automated data-dependent acquisition (DDA) method with collision energy ramped to 35 eV, fragmenting up to five non-targeted precursor ions per scan. Mzmine 3.9.0 was utilized for processing the acquired MS data.

### 2.3 Screening the key targets of PUR

The active ingredients in PUR were searched in the TCMSP database (https://tcmsp-e.com/tcmsp.php) using "Gegen" as the keyword, and were screened according to the criteria of oral bioavailability (OB) ≥ 10% and drug similarity DL ≥ 0.18 11. The active components of PUR were input into the TCMSP and PubChem (https://pubchem.ncbi.nlm.nih.gov/) databases to identify drug targets. The corresponding genes of the target proteins were queried in the UniProt (https://www.uniprot.org/) database and sorted. After removing duplicated genes, we obtained 218 targets for further analysis.

### 2.4 Screening of active ingredients in liver fibrosis and construction of ingredient network

Using the keywords "liver fibrosis" and " hepatic fibrosis," we collected disease-related targets from the Genecards database (https://www.genecards.org/) and Online Mendelian Inheritance in Man (OMIM) database (http://www.omim.org). The targets were combined and duplicate genes and false-positive genes were removed to get liver fibrosis targets. The collected liver fibrosis targets are matched with the PUR active ingredient targets, and the resulting common targets are the potential liver fibrosis inhibition targets of PUR. To further investigate the interactions among functional components, targets, and signaling pathways, PPI network was then constructed using STRING 12.0 (https://string-db.org/) and Cytoscape (version 3.7.2). The filtered analysis files were imported into Cytoscape 3.7.2, and the PPI network diagram was visualized.

### 2.5 KEGG and GO pathway enrichment analyses

Gene ontology (GO) functional enrichment analysis was performed using the Database for Annotation, Visualization and Integrated Discovery (DAVID, https://david.ncifcrf.gov/) to investigate cellular composition (CC), molecular function (MF) and biological Process (BP). Furthermore, pathway enrichment analysis was performed using the Kyoto Encyclopedia of Genes and Genomes (KEGG) pathway repository (https://david.ncifcrf.gov/) 12-14 to elucidate pathways associated with the PUR effect. Adjusted *p* -value ≤ 0.0001 and count ≥ 10 were selected in the functional annotation clustering.

### 2.6 Molecular docking

Molecular docking is a valuable computational tool for predicting the potential therapeutic effects of drug components by evaluating their binding affinities with target proteins, particularly hub genes. In this study, molecular docking was performed using AutoDock 4.2 to estimate the binding affinities between key active components and their respective target receptors. The resulting docked complexes were visualized using Discovery Studio 2021, providing detailed insights into the binding interactions, including hydrogen bonding, hydrophobic interactions, and the involvement of specific amino acid residues within the binding site.

### 2.7 Animal experiment

Male C57BL/6 mice (5 weeks) were obtained from the National Laboratory Animal Center (Taipei, Taiwan). The mice were acclimated to the laboratory environment for one week under a 12-h light/dark cycle at 23 °C, with free access to food and water. All experimental procedures were conducted in accordance with the National Institutes of Health (NIH) guidelines for the care and use of laboratory animals. All animal research procedures were reviewed and approved by the Taipei Medical University Institutional Animal Care and Use Committee (IACUC) (License No. LAC-2022-0137). All methods are reported in accordance with the ARRIVE guidelines (https://arriveguidelines.org). Liver fibrosis was induced by intraperitoneal injection of carbon tetrachloride (CCl₄) diluted 1:1 in olive oil at a dose of 2 mL/kg, administered twice per week for 4 consecutive weeks. After model establishment, the mice were randomly divided into four groups (n = 6 per group): CCl₄ group, PUR-L group (125 mg/kg PUR50E), PUR-H group (500 mg/kg PUR50E), and a positive control group treated with silymarin (50 mg/kg). A vehicle control group received olive oil only without CCl₄ and was gavaged daily with ddH₂O. All other groups continued to receive CCl₄-olive oil injections to maintain liver fibrosis and were simultaneously administered the respective treatments by oral gavage for an additional 2 weeks. At the end of the experiment, the mice were euthanized by carbon dioxide (CO₂) inhalation in accordance with the **AVMA Guidelines for the Euthanasia of Animals: 2020 Edition**. The CO₂ flow rate was maintained at 30-70% of the chamber volume per minute until respiration ceased. Liver tissues and serum samples were then collected and stored at -80 °C for subsequent analyses. Previous studies have used crude extracts of *Pueraria lobata* or pure puerarin at doses ranging from 100 to 600 mg/kg in animal models of metabolic and liver diseases, demonstrating favorable bioactivity and tolerability within this range 15-17.

### 2.8 Biochemical analysis

After blood collection, plasma was extracted immediately by centrifugation at 4000 rpm for 20 min. AST, ALT, TGF-β1 were assayed with an automatic biochemistry analyzer from Roche.

### 2.9 Histology and immunohistochemistry

Liver tissues were collected and processed for histological analysis. The tissues were fixed in 10% formaldehyde solution, followed by paraffin embedding and sectioning at 5 μm thickness. For morphological assessment, sections were stained with hematoxylin and eosin (H&E), Masson's trichrome, and Sirius red. For immunohistochemical analysis, sections underwent deparaffinization and antigen retrieval before overnight incubation with primary antibodies at 4 °C. The antibodies used were anti-α-SMA (1:200 dilution, Proteintech) and anti-Fibronectin (1:100 dilution, Santa Cruz). Immunohistochemical staining was then performed according to the manufacturer's protocol. All stained sections were examined and photographed using a light microscope.

### 2.10 Western Blotting Assay

Liver tissues were lysed using RIPA buffer, and the lysates were centrifuged at 12,000 rpm for 30 minutes to collect the supernatant containing total protein. Protein concentrations were determined using the Bradford assay. Western blot analysis was then conducted using specific primary antibodies and appropriate HRP-conjugated secondary antibodies, following a previously established protocol. The antibodies employed in this study included Fibronectin (Santa Cruz, sc-8422), Collagen Type I (Proteintech, 14695-1-AP), α-SMA (Proteintech, 14395-1-AP), MMP-3 (Santa Cruz, sc-21732), MMP-8 (Santa Cruz, sc-8848), MMP-13 (Cell Signaling, #94808), Nrf2 (Cell Signaling, #20733), and HO-1 (Cell Signaling, #26416). The intensity of protein bands was quantified using ImageJ software, and the expression levels of target proteins were normalized to GAPDH as the internal control.

### 2.11 Quantitative real-time PCR (qPCR) analysis

Total RNA was extracted from liver tissue using Trizol reagent (Sigma) and reverse transcribed into cDNA using iScript™ cDNA Synthesis Kit (Bio-Rad). qRT-PCR was performed using ORA™ SEE qPCR Green ROX L Mix, 2X (highQu). Data collection was performed by means of a LightCycler®480 system (Roche). The expression level of GAPDH mRNA was used to normalize the data of desired gene using 2-ΔΔCt method. The sequence of primers was listed in Table S1.

### 2.12 Metabolomics profiling

Metabolites were extracted from plasma samples using a cold solvent mixture of methanol/acetonitrile/water (2:2:1, v/v/v). Briefly, 500 μl of extraction solvent was added to 120 mg plasma and thoroughly vortexed. The mixture was incubated on ice for 20 minutes, followed by centrifugation at 14,000 x g for 20 minutes at 4 °C. The resulting supernatant was collected and dried using a vacuum centrifuge at 4 °C. Prior to LC-MS analysis, the dried extracts were reconstituted in 100 μl of acetonitrile:water (1:1, v/v) and transferred to LC autosampler vials. Metabolomic analysis was performed using Ultra-High Performance Liquid Chromatography-Tandem Mass Spectrometry (UHPLC-MS/MS) 18, 19. The metabolomic data were subjected to multivariate statistical analysis, including Principal Component Analysis (PCA) and Orthogonal Projections to Latent Structures Discriminant Analysis (OPLS-DA) (Tang et al., 2023). Differential metabolites were identified using Variable Importance in Projection (VIP) scores and S-plots. Metabolites with VIP scores > 1.0 and p-values < 0.05 were considered as potential biomarkers. The fundamental data analysis comprised both univariate and multivariate statistical analyses of the metabolomic profiles 20, enabling the identification of significantly altered metabolites.

### 2.13 Statistical Analyses

Statistical analyses were performed using GraphPad Prism 8.0 (GraphPad Software Inc., San Diego, CA, USA). Results are presented as mean ± SD. The Kolmogorov-Smirnov test was applied to verify the normal distribution of all continuous variables. Differences between multiple experimental groups were evaluated using one-way ANOVA, with subsequent Tukey's post hoc test for pairwise comparisons. A *p*-value < 0.05 was established as the threshold for statistical significance.

## 3. Results

### 3.1 Identification of Components in PUR50E by HPLC-QTOF-MS/MS

The constituents were first identified in PUR50E by HPLC-QTOF-MS/MS. The positive ion mode and negative ion mode chromatogram is shown in Figure S1. A total of 24 compounds as show in Table 1. These compounds represent the material basis of PUR50E in improving liver fibrosis. Quantitative analysis was performed for puerarin, which was selected as the marker compound for standardization of PUR50E. Puerarin was quantified using LC-MS with an external standard calibration curve, showing excellent linearity (R² = 0.9999) over the concentration range of 7.8125-125 μg/mL. The content of puerarin in PUR50E was determined to be 44.29 mg/g extract. In addition, LC-MS based chemical fingerprint analysis was conducted to establish a quality control profile of PUR50E. A major peak at RT 19.143 min corresponded well with the puerarin reference standard (RT 19.119 min), confirming puerarin as a predominant constituent. This fingerprint chromatogram serves as a standardization indicator to ensure batch-to-batch consistency of the extract (Figure S2).

### 3.2 Network pharmacology analysis and molecular docking validation of PUR50E against TGF-β1 in liver fibrosis

**Figure 1:**
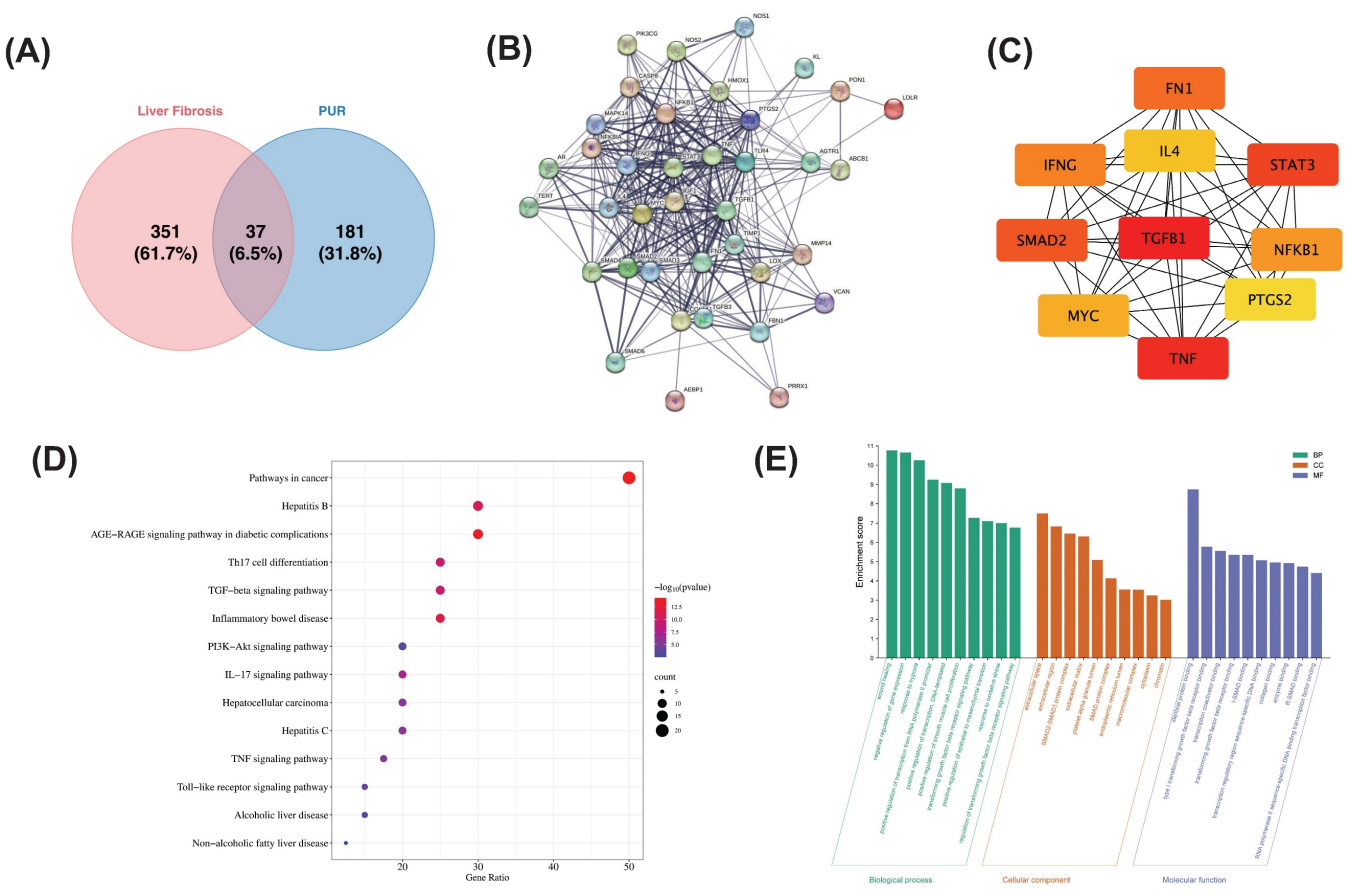
** Network pharmacology analysis of the potential mechanism of PUR in treating liver fibrosis. (A)** Venn diagram of intersection genes of active ingredient related targets of PUR and liver fibrosis targets. **(B)** PPI network of PUR in the treatment of liver fibrosis. **(C)** The identification of ten hub genes from the protein-protein interaction (PPI) network was conducted using the maximal clique centrality (MCC) algorithm. **(D)** KEGG pathway analysis. **(D)** GO enrichment analysis.

Network pharmacology was utilized to investigate the mechanism of PUR50E in treating liver fibrosis. Initially, 388 liver fibrosis-related target genes were identified from the GeneCards and OMIM databases after deduplication. Subsequently, 218 potential targets related to PUR were identified using the PubChem and TCMSP databases. Intersection of these datasets revealed 37 targets associated with both PUR and liver fibrosis (Fig. 1A). To analyze the protein-protein interaction (PPI) relationships among these targets, the STRING database was used to generate a PPI network (Fig. 1B), where nodes represent target proteins and edges represent interactions. In this network, higher degree centrality indicates stronger relationships between proteins, suggesting greater importance of a target protein in the network. Using the CytoHubba algorithm, the top 10 hub genes were identified as follows: TGFβ1, TNF, STAT3, AMAD2, FN1, IFNG, NFKβ1, MYC, IL4, and PTGS2 (Fig. 1C).

KEGG pathway analysis revealed that these targets are primarily involved in pathways such as pathway in cancer, TGF-β1 signaling pathway, IL-17 signaling pathway and PI3K-AKT signaling pathway (Fig. 1D). Additionally, GO enrichment analysis indicated that in the BP category, the targets are involved in processes such as wound healing, negative regulation of gene expression, positive regulation of RNA polymerase II promoter transcription, positive regulation of smooth muscle cell proliferation, regulation of the TGF-β receptor signaling pathway, positive regulation of epithelial-mesenchymal transition, response to oxidative stress, and regulation of the TGFβ receptor signaling pathway. In the MF category, the targets exhibit activities such as protein-protein binding, enzyme binding, and sequence-specific DNA binding for transcriptional regulation. In the CC category, the targets are predominantly located in the cytoplasm, extracellular space, and extracellular regions (Fig. 1E). These results suggest that the active pharmacological components in PUR may act on the TGF-β1 signaling pathway and oxidative stress to alleviate the progression of liver fibrosis.

**Figure 2:**
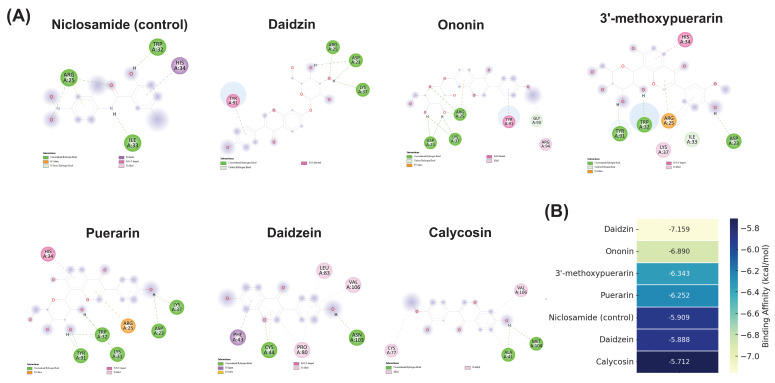
** Molecular docking interactions and binding affinities of major flavonoids from PUR with TGF-β1. (A)** 2D interaction diagrams showing hydrogen bonds and hydrophobic contacts between niclosamide (control), puerarin, daidzein, daidzin, 3'-methoxypuerarin, ononin, and calycosin with TGF-β1. **(B)** Heatmap of binding affinities (kcal/mol) of major flavonoids in PUR and niclosamide (control) to TGF-β1 receptor. Lighter colors indicate stronger binding. Daidzin and ononin exhibited the strongest binding affinities.

Based on the results of network pharmacology, TGF-β1 was identified as one of the key hub targets associated with liver fibrosis. To further confirm the potential interaction between key compounds in PUR and TGF-β1, molecular docking was performed. As shown in Figure 2 A and B, all six major flavonoids exhibited stable binding affinities with TGF-β1, with daidzin (-7.159 kcal/mol) and ononin (-6.890 kcal/mol) showing the strongest interactions. These binding energies were more favorable than that of the positive control niclosamide (-5.909 kcal/mol), suggesting a potential inhibitory effect of these compounds on TGF-β1-mediated fibrogenic signaling.

### 3.3 Effects of PUR50E on Liver Fibrosis and Collagen Deposition in CCl_4_-Induced Mice

**Figure 3:**
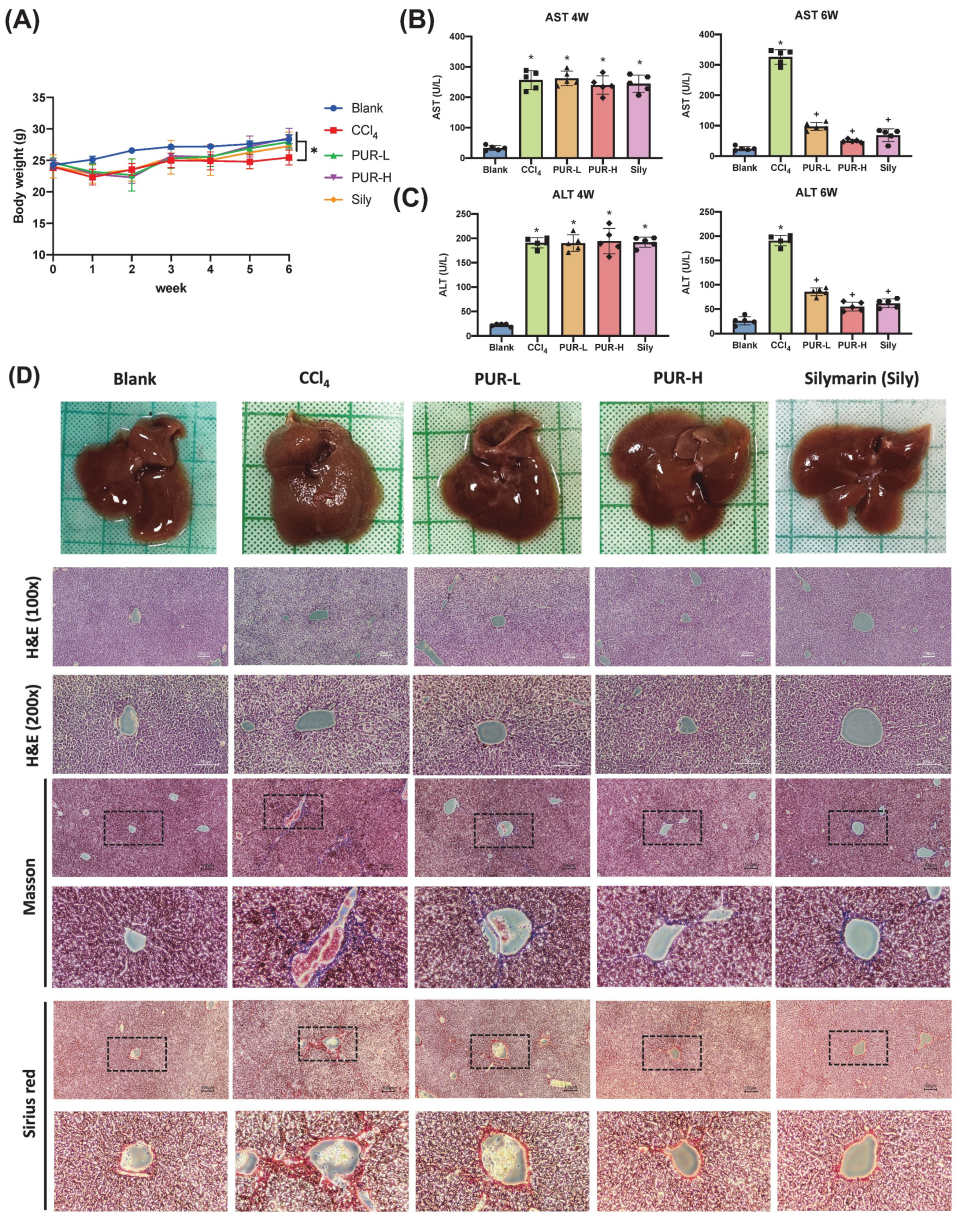
** PUR50E attenuates CCl₄-induced liver injury and collagen fiber accumulation in mice. (A)** Weekly recording of mouse body weight. **(B, C)** Plasma levels of AST and ALT measured at weeks 4 and 6. **(D)** Liver morphology and histopathology results (H&E, Masson, and Sirius red staining). * *p*<0.05, significant difference compared to the blank group; + *p*<0.05, significant difference compared to the CCl₄ group.

To investigate the role of PUR50E in liver fibrosis development, we established a chronic liver fibrosis mouse model using CCl_4_. Silymarin (Sily) was selected as a positive control to evaluate the therapeutic effects of PUR50E. During the first week of CCl_4_ induction, the body weight of mice significantly decreased compared to the Blank group. Starting from the fourth week, after oral administration of PUR50E, body weight noticeably recovered (Fig. 3A). Four weeks of CCl_4_ induction resulted in a significant increase in plasma AST and ALT levels in mice (Fig. 3B). After PUR50E treatment, serum AST and ALT levels were significantly reduced (Fig. 3C). Observations of liver tissue morphology and histopathological staining showed that, compared to the Blank group, mice in the CCl_4_ group exhibited severe liver damage, with a rough liver surface texture and noticeable granularity. In contrast, mice in the PUR-L and PUR-H groups showed reduced liver surface roughness and granularity, presenting a smooth surface similar to that of the Blank group. H&E staining results revealed that, compared to the Blank group, liver tissue sections from the CCl_4_ group displayed loose structures, decreased cell density, and increased vacuolization. After PUR50E treatment, tissue structures were more organized, resembling those of the Blank group. Masson staining indicated a significant increase in collagen fiber deposition (blue) in the CCl_4_ group, which was reduced following PUR50E treatment. Similarly, Sirius Red staining showed a marked increase in collagen fiber deposition (bright red) in the CCl_4_ group, which was also reduced after PUR50E treatment (Fig. 3D).These results suggest that PUR50E effectively reverses CCl4-induced liver collagen fiber accumulation, with outcomes similar to those observed in the Silymarin-treated group.

### 3.4 Effects of PUR50E on Collagen-Related mRNA expression in CCl_4_-Induced Mice

**Figure 4:**
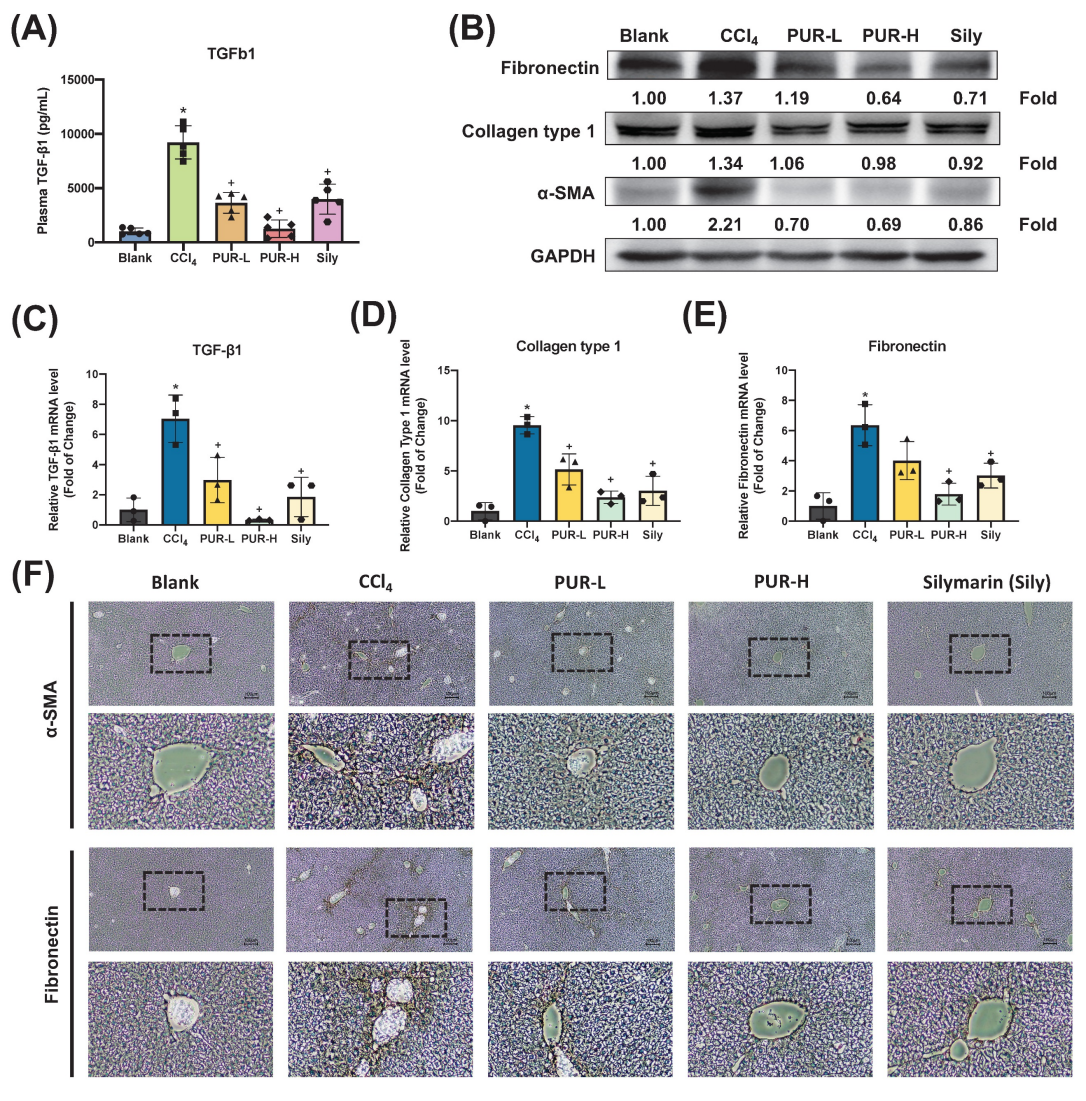
** Effect of PUR50E on fibrosis-related markers in CCl₄-induced liver fibrosis. (A)** Plasma levels of TGF-β1 in mice. **(B)** Western blot analysis of Fibronectin, Collagen Type I, and α-SMA protein expression. qPCR analysis of mRNA expression in liver tissue for **(C)** TGF-β1, **(D)** Collagen Type I, and **(E)** Fibronectin. **(F)** Immunohistochemistry analysis of α-SMA and Fibronectin protein expression in liver tissue sections. * *p*<0.05, significant difference compared to the blank group; + *p*<0.05, significant difference compared to the CCl₄ group.

To further validate the anti-fibrotic effects of PUR50E, plasma TGF-β1 levels were measured and found to be reduced by PUR50E in CCl_4_-induced mice (Fig. 4A). qPCR analysis revealed that PUR50E reduced mRNA expression of pro-fibrotic factors (TGF-β1, Collagen Type I, and Fibronectin) compared to the CCl_4_-induced group (Fig. 4B-C). Immunohistochemical (IHC) staining further confirmed decreased expression of α-SMA and Fnin the PUR50E treatment groups (Fig. 4D). The findings demonstrate that PUR50E significantly alleviates CCl₄-induced liver fibrosis by suppressing collagen-associated protein and gene expression, showing efficacy similar to the Sily group.

### 3.5 Effects of PUR50E on MMPs Expression and Antioxidant Activity in CCl_4_-Induced Mouse Liver

**Figure 5:**
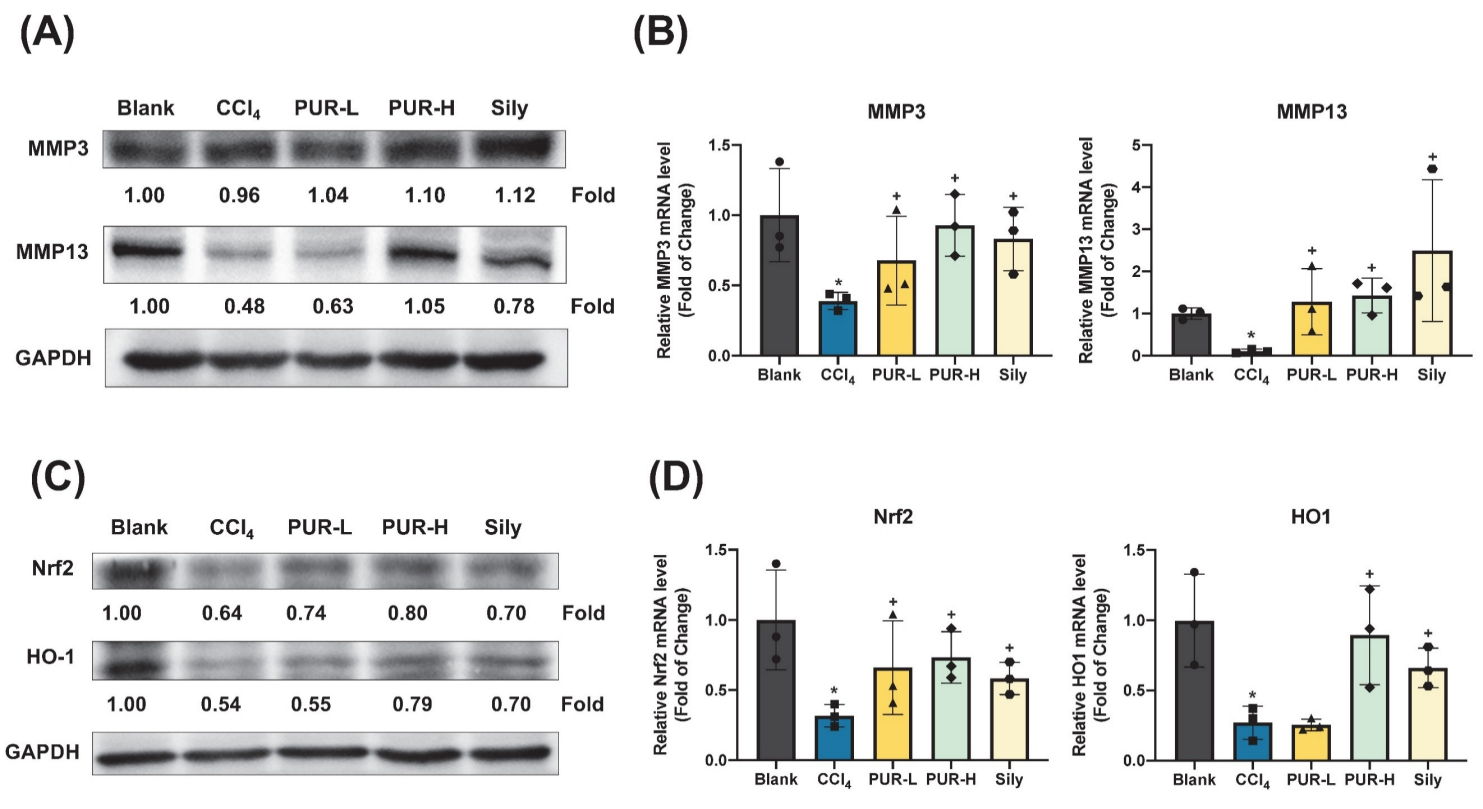
** PUR50E increased the expression of MMPs and enhances Nrf2/HO-1 protein and mRNA levels in the CCl₄-induced mouse liver. (A)** Western blot analysis of MMP3 and MMP13 protein expression. **(B)** qPCR analysis of mRNA expression in liver tissue for MMP3 and MMP13. **(C)** Western blot analysis of Nrf2 and HO-1 protein expression. **(D)** qPCR analysis of mRNA expression levels for Nrf2 and HO-1 in liver tissue * *p*<0.05, significant difference compared to the blank group; + *p*<0.05, significant difference compared to the CCl₄ group.

MMPs play a key role in alleviating liver fibrosis by degrading extracellular collagen. Western blot analysis revealed that PUR50E treatment partially restored the expression of MMP3 and MMP13 in liver tissues of CCl_4_-induced mice, with MMP13 showing the most notable increase (Fig. 5A). Moreover, qPCR analysis indicated that the mRNA levels of MMP3 and MMP13 were significantly higher in the PUR50E treatment group compared to the CCl_4_-induced group (Fig. 5B), suggesting that PUR50E promotes MMP expression to mitigate liver fibrosis. Network pharmacology analysis suggested that PUR50E may mitigate liver fibrosis by modulating oxidative stress, a key driver of fibrosis progression. Oxidative stress induces DNA and protein oxidation, as well as lipid peroxidation in hepatocytes, leading to cellular damage and apoptosis. To explore PUR50E's antioxidative mechanism, we examined the expression of the antioxidant transcription factor Nrf2 and its downstream effector HO-1. In the CCl_4_-induced group, Nrf2 and HO-1 protein and mRNA levels were significantly reduced. However, PUR50E treatment markedly enhanced the expression of Nrf2 and HO-1 (Fig. 5C-D), indicating that PUR50E activates the Nrf2 signaling pathway to protect the liver from oxidative stress. Collectively, these findings demonstrate that PUR50E improves CCl_4_-induced liver fibrosis by promoting MMP expression to degrade extracellular collagen and by activating the Nrf2 signaling pathway to counteract oxidative stress.

### 3.6 Metabolomic analysis of the effects of PUR50E on liver fibrosis in mice

Serum samples were analyzed using PCA, and the samples were plotted based on their principal component scores in a 2D plane. In both ESI^+^ and ESI^-^ modes, clear separation was observed between the clusters of the Blank group and the Control group, as well as between the Control group and the High-dose group (Figure S3 A). Additionally, the clusters of the Blank group and the High-dose group appeared to be closer to each other. An interactive 3D scatter plot was also generated using the R package **plotly** (v4.9.3), allowing rotation to observe the distribution and distinction among samples from a more dimensional perspective by simultaneously examining three principal components (Figure S3B). Biplots of PCA performed on the metabolites identified in mouse serum. The top 30 features contributing to PC1 and PC2 in the PCA plot were identified (Figure S3C).

**Figure 6:**
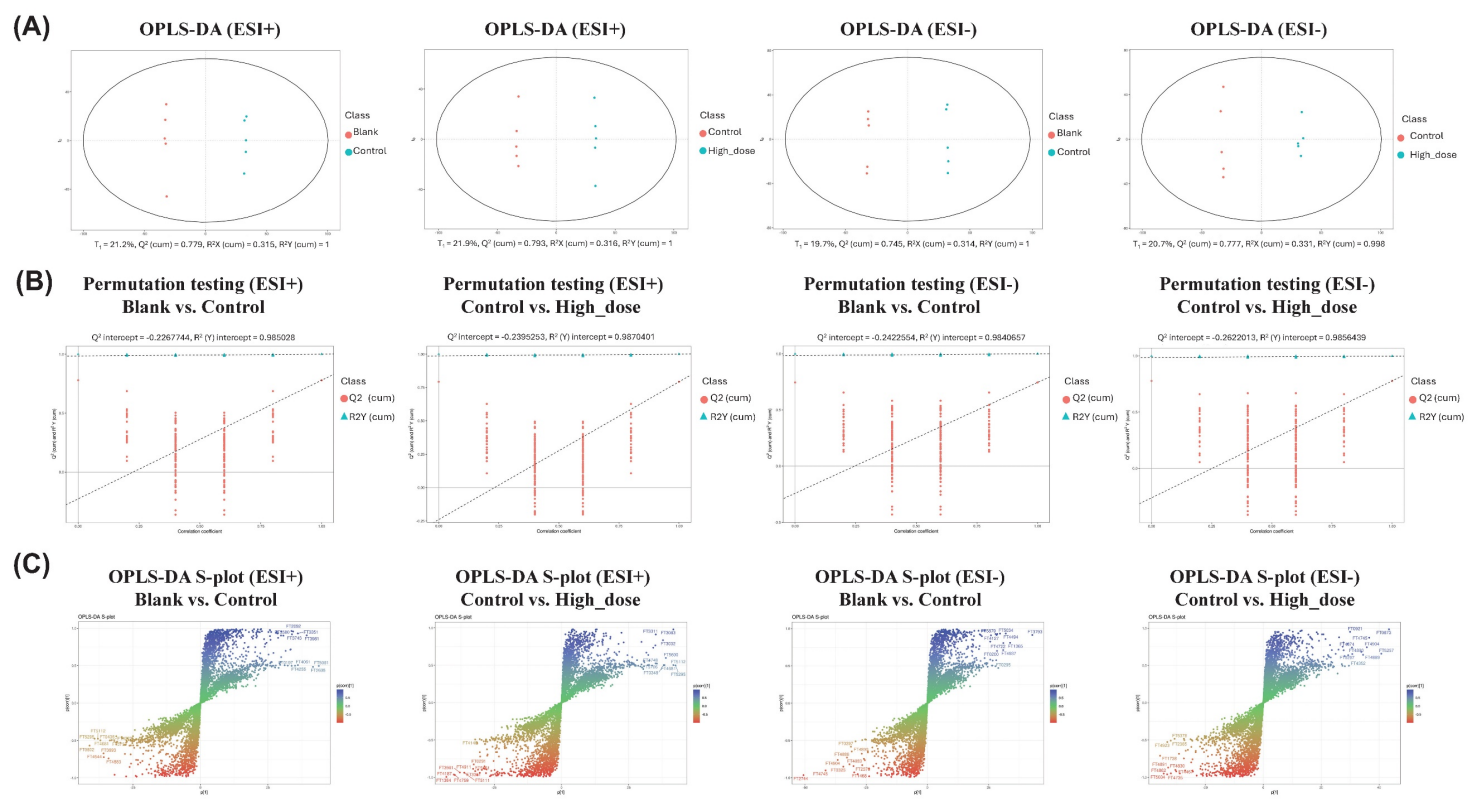
** OPLS-DA score plots, S-plots and validation analysis of metabolomic data in positive and negative ion modes. (A)** OPLS-DA score plots demonstrate metabolic profile differences between Blank versus Control groups and Control versus High-dose groups in both ESI^+^ and ESI^-^ modes. **(B)** Model validation using 999-permutation tests for OPLS-DA analyses comparing Blank versus Control groups and Control versus High-dose groups in both ESI^+^ and ESI^-^modes. **(C)** S-plots derived from OPLS-DA analysis revealing metabolite distribution patterns between Blank versus Control groups and Control versus High-dose groups under both ESI^+^ and ESI^-^ conditions.

The OPLS-DA results demonstrated robust model performance across both ESI^+^ and ESI^-^ modes in serum samples. For ESI^+^ mode, the analysis of blank versus control groups yielded values of R^2^X=0.315, R^2^Y=1, and Q^2^ =0.779, while control versus high-dose groups showed R^2^X=0.316, R^2^Y=1, and Q^2^=0.793. Similarly, in ESI^-^ mode, blank versus control groups demonstrated R^2^X=0.314, R^2^Y=1, and Q^2^=0.745, with control versus high-dose groups showing R^2^X=0.331, R^2^ Y=0.998, and Q^2^ =0.777. These parameters indicated good explanatory and predictive capabilities of the models (Fig. 6A). Through 999 iterations of permutation testing, the results indicate that the R^2^ value is greater than Q^2^, and the intercept of Q^2^ with the y-axis is less than 0, confirming the absence of overfitting and establishing the models' validity and reliability (Fig. 6B). The S-plot analysis was employed to identify discriminating features between groups, where the horizontal axis represented the variation magnitude of measured variables within the modeled covariation, and the vertical axis indicated reliability (modeled correlation). Features positioned at the top right or bottom left of the plot were identified as the most significant discriminating variables (Fig. 6C). To achieve a comprehensive characterization of differential metabolites, the potential biomarkers identified from both ESI^+^ and ESI^-^ modes were combined for subsequent analyses.

### 3.7 Metabolic pathway analysis

**Figure 7:**
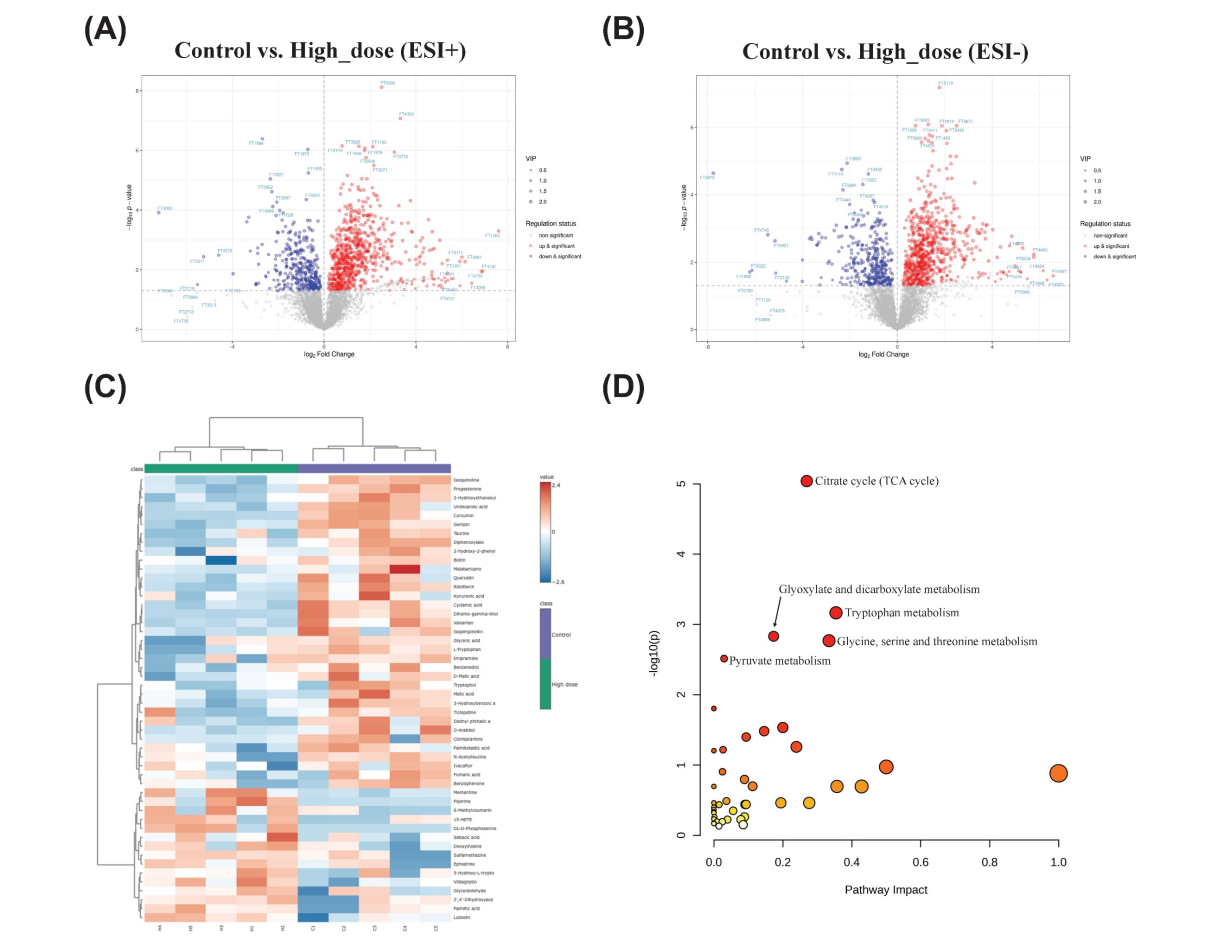
** Metabolites change and the associated pathway in mouse serum. Volcano plot of differentially expressed metabolites. (A)** Control and High_dose group (ESI^+^), **(B)** Control and High_dose group (ESI^-^), red indicates upregulation, blue indicates downregulation, and non-significantly different metabolites are in gray. The size of the dot represents the VIP value, the larger the dot, the larger the VIP value. **(C)** Heatmaps of the top 50 differential serum metabolites in Control and High_dose groups. **(D)** Pathways significantly influenced in serum samples between the Control group and High_dose group, including TCA cycle, tryptophan metabolism, glyoxylate and dicarboxylate metabolism, glycine, serine and threonine metabolism and pyruvate metabolism.

**Figure 8:**
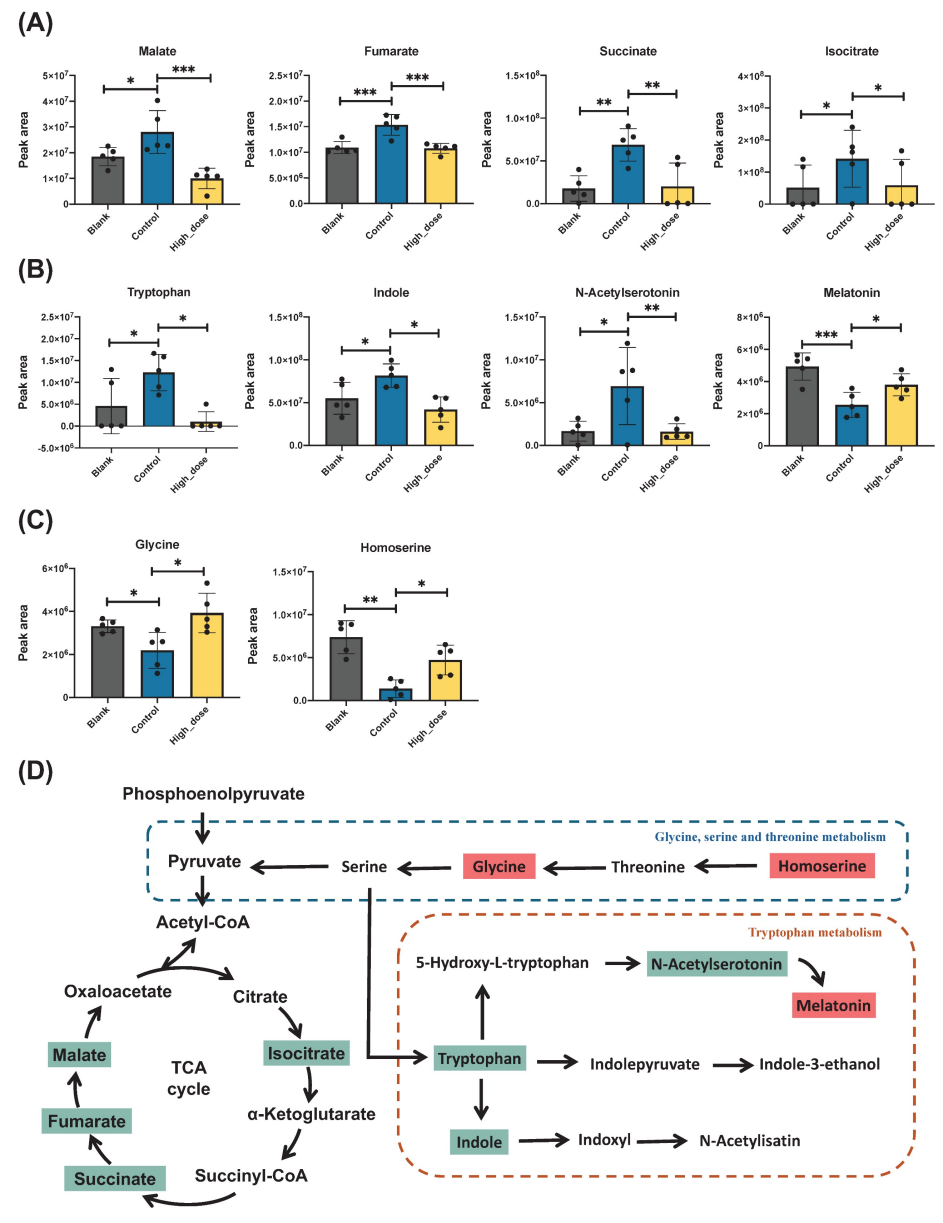
** Potential metabolites changes in TCA cycle and tryptophan metabolism. Peak intensities of (A)** TCA cycle-related metabolites, **(B)** tryptophan metabolic pathway and **(C)** Glycine, serine and threonine metabolism. **(D)** Metabolic signaling networks showing differentially regulated pathways. Red boxes represent metabolites with significant upregulation, while green boxes indicate significantly downregulated metabolites in the PUR50E treatment groups compared to control groups. * *p*<0.05 and ** *p*<0.01 significant difference compared to the Control group.

The differentially expressed genes are shown in a volcano plot. In the positive ion mode, compared with the control group, 54 metabolites increased and 51 metabolites decreased in the high-dose metabolite group (Fig. 7A). In the negative ion mode, 28 metabolites increased and 39 metabolites decreased (Fig. 7B). A total of 164 differential metabolites were identified in positive and negative ion modes, and a heat map was constructed to show the intensity levels of the top 50 differential metabolites between the two groups (Fig. 7C). To explore the mechanism of action of PUR50E in liver fibrosis, the identified potential metabolites were imported into MetaboAnalyst 6.0 for metabolic pathway analysis. The top 3 functions involved in Citrate cycle (TCA cycle), Tryptophan metabolism and Glyoxylate and dicarboxylate metabolism (Fig. 7D). We analyzed specific changes in serum metabolites in these three metabolic pathways. PUR50E treatment decreased the levels of malate, fumarate, succinate and isocitrate in the TCA cycle compared to control groups (Fig. 8A). In the tryptophan metabolism pathway, PUR50E reduced the levels of tryptophan, indole, and *N*-acetylserotonin, while increasing melatonin levels compared to control groups (Fig. 8B).In glyoxylate and dicarboxylate metabolism pathway PUR50E upregulate glycine and homoserine levels compared to control groups (Fig. 8C). Based on the above results, we constructed signaling networks associated with the differentially expressed metabolic pathways (Fig. 8D). These metabolic changes demonstrate that PUR50E regulates TCA cycle, tryptophan metabolism and glyoxylate and dicarboxylate metabolism, thereby improving liver energy metabolism and reversing the development of liver fibrosis. In addition, the observed alterations in these metabolites may serve as potential diagnostic biomarkers for liver fibrosis.

## 4. Discussion

Liver fibrosis is a key pathological feature of many chronic liver diseases. Although potentially reversible, uncontrolled fibrosis can progress to cirrhosis and hepatocellular carcinoma. Currently, effective pharmacological interventions remain limited. Natural compounds, with their low toxicity, minimal side effects, and high biological activity, have gained increasing attention in fibrosis research 21-23. In this study, we integrated network pharmacology and metabolomics to elucidate the anti-fibrotic mechanisms of PUR50E, a 50% ethanol extract of *Pueraria lobata* (Willd.) Ohwi. Network pharmacology analysis identified TGF-β1 as a key hub target, which was further validated by molecular docking showing strong binding affinities of PUR50E flavonoids. *In vivo* experiments demonstrated that PUR50E significantly attenuated CCl₄-induced liver fibrosis in mice through multiple mechanisms, including suppression of ECM deposition, modulation of MMP activity, activation of the Nrf2/HO-1 antioxidant pathway, and regulation of tryptophan metabolism and the TCA cycle. Collectively, these findings underscore the multi-target, multi-pathway therapeutic potential of PUR50E and support its further development for the treatment of liver fibrosis.

Network pharmacology analysis revealed that PUR50E exerts anti-fibrotic effects through multi-target and multi-pathway mechanisms. Intersection analysis identified 37 overlapping targets between PUR and liver fibrosis, with TGF-β1 emerging as a central hub in the PPI network. KEGG pathway enrichment suggested the involvement of the TGF-β signaling pathway, IL-17 signaling, PI3K-Akt signaling, and cancer-related pathways, while GO analysis highlighted roles in wound healing, oxidative stress regulation, and epithelial-mesenchymal transition. These findings indicated that TGF-β1-mediated signaling and oxidative stress regulation may be critical mechanisms for PUR50E in alleviating liver fibrosis.

In the present study, molecular docking was performed to further validate the network pharmacology predictions and to explore the potential interactions between the major active flavonoids of PUR and TGF-β1, a central mediator of hepatic fibrosis. The results revealed that daidzin (-7.159 kcal/mol) and ononin (-6.890 kcal/mol) exhibited the strongest binding affinities, both surpassing the positive control niclosamide (-5.909 kcal/mol), followed by 3'-methoxypuerarin, puerarin, daidzein, and calycosin. This suggests that these compounds may directly interact with TGF-β1 and potentially interfere with its ligand-receptor engagement, thereby attenuating downstream activation of hepatic stellate cells and extracellular matrix deposition. Interestingly, glycosylated derivatives (e.g., daidzin and ononin) displayed stronger binding affinities compared to their aglycone counterparts (daidzein and calycosin), indicating that the sugar moieties may enhance hydrogen bonding and overall binding stability within the TGF-β1 binding pocket. This observation aligns with previous reports that certain glycosylated isoflavones exhibit improved receptor interactions and bioavailability 24. Consistent with earlier studies showing that puerarin and structurally related isoflavones can inhibit TGF-β/Smad signaling in liver fibrosis models 25, our docking analysis provides structural evidence supporting their direct interaction with TGF-β1. These findings integrate network pharmacology predictions with molecular docking validation, suggesting that PUR50E may exert its anti-fibrotic effects, at least in part, through direct modulation of TGF-β1 signaling.

Liver fibrosis is a complex pathological process characterized by excessive ECM deposition, primarily driven by collagens and fibronectin. ECM components, such as Col-I, Col-III, Fibronectin and α-SMA, are deposited in large quantities within the liver lobules, leading to structural destruction and liver dysfunction. Fibronectin, a multifunctional glycoprotein, is synthesized by various cell types including fibroblasts, endothelial cells, macrophages, and hepatocytes 26. Its expression correlates strongly with liver fibrosis progression and extracellular matrix accumulation, serving as a key biomarker for hepatic fibrosis assessment 27. Our findings demonstrate that PUR50E reduces mRNA expression of α-SMA and fibronectin. Furthermore, these experimental findings support our network pharmacology prediction analysis.

MMPs, such as MMP-3 and MMP-13, play critical roles in ECM remodeling, while their dysregulation contributes to fibrosis progression. MMP-3 functions as a key enzyme capable of degrading numerous ECM components, such as collagens (types II, III, and IV), elastin, fibronectin, laminin, and proteoglycan. Furthermore, MMP-3 acts as a crucial mediator in the liver by facilitating the activation cascade of other MMPs, including MMP-1, MMP-7, and MMP-9. 28. While MMP-3 can facilitate ECM remodeling, it has also been implicated in promoting fibrosis by enhancing the activity of other profibrotic factors, particularly in chronic liver injury contexts 29. MMP-13 is specifically effective at degrading collagen types I and II and is crucial for normal tissue remodeling 30, 31. Although MMP-13 has important antifibrotic effects, its expression level may vary at different stages of the disease, so it is necessary to carefully consider whether increasing its activity can effectively reduce liver fibrosis 32. In our CCl_4_-induced liver fibrosis model, we observed a significant decrease in MMP-3and MMP-13 expression, leading to ECM accumulation. PUR50E treatment restored these MMPs. Increasing these enzymes' activity presents a promising strategy for mitigating liver fibrosis by promoting ECM degradation. However, a thorough understanding of their regulatory mechanisms and potential side effects is crucial for developing effective treatments. Further research is needed to elucidate their roles in different stages of liver disease and to refine therapeutic strategies targeting these enzymes.

Oxidative stress represents a critical mechanism underlying liver damage and fibrosis initiation. It emerges from a disrupted equilibrium between cellular pro-oxidant and antioxidant systems, characterized by increased production of reactive oxygen species (ROS) and reactive nitrogen species (RNS). These pro-fibrotic mediators, which include superoxide, hydrogen peroxide (H_2_O_2_), and hydroxyl radicals, play a pivotal role in driving pathological liver transformation 33. The Nrf2/HO-1 pathway has emerged as a significant player in the context of liver fibrosis. Oxidative or electrophilic stress triggers the release of Nrf2 from its cytoplasmic inhibitor Keap1, allowing Nrf2 to enter the nucleus where it binds to ARE sequences, activating the expression of antioxidant enzymes including HO-1, CAT, and SOD. Natural compounds have been shown to enhance Nrf2 expression, thereby increasing HO-1 levels and alleviating liver damage and fibrosis 34, 35. In a CCl_4_-induced liver fibrosis mouse model, PUR50E demonstrated the ability to upregulate mRNA expression of the Nrf2/HO-1 pathway. By mitigating oxidative damage, the compound effectively suppressed liver fibrosis progression. These findings suggest potential antioxidant and hepatoprotective mechanisms of PUR50E, which is consistent with the predictions of network pharmacology.

In the CCl_4_-induced liver fibrosis model, PUR50E demonstrated significant regulatory effects on hepatic metabolic pathways. Metabolomic analysis revealed that TCA cycle, Tryptophan metabolism and Glycine, serine and threonine metabolism were the top 3 metabolic pathways affected by PUR50E treatment. TCA cycle dysfunction is typically associated with disrupted cellular energy metabolism, increased oxidative stress, and cell death 36. Clinical metabolomics studies have further identified elevated malate, fumarate, and succinate in serum samples from patients with HCC and HBV-related cirrhosis 37 , while cohort analyses in NAFLD and NASH have associated enhanced TCA cycle activity with fibrosis progression, particularly in male patients 38.

Consistent with these findings, our study demonstrated significant elevations in serum levels of malate, fumarate, succinate, and isocitrate in CCl₄-induced fibrotic mice compared with healthy controls. Importantly, PUR50E treatment markedly reduced the abundance of these metabolites, suggesting that modulation of TCA cycle intermediates may represent not only a therapeutic outcome of PUR50E but also potential metabolic targets for the treatment of liver fibrosis.

Tryptophan metabolism plays crucial roles in various physiological processes, including immune responses, fibrosis, glucose control, lipid metabolism, and hormonal homeostasis 39. In CCl_4_-induced liver fibrosis, dysregulation of the Tryptophan metabolic pathway may be associated with inflammatory responses, oxidative stress, and hepatocellular injury. Interestingly, a previous study found that tryptophan treatment further increased the expression of TGF-β and pancreatin α1(I) in the hepatopancreas of high fat and high fructose diet-induced mice and also showed an increase in serum ALT levels and the formation of ROS 40. In our study, we also found that PUR50E could inhibit tryptophan metabolites expression in CCL_4_ -induce mice.

Glycine deficiency is a hallmark of fatty liver and is linked to liver fibrosis, with low glycine levels impairing glutathione (GSH) synthesis and increasing oxidative stress 41. A large cross-sectional study demonstrated that serum glycine levels in patients with liver fibrosis were significantly lower than those in the normal population 42. Homoserine, a derivative of glycine, has not yet been directly associated with liver fibrosis, although its involvement in amino acid and energy metabolism suggests a potential indirect role. Interestingly, in this study, both glycine and homoserine metabolites were significantly decreased in mice with CCl₄-induced liver fibrosis, which is consistent with previous reports of glycine deficiency in chronic liver injury. Importantly, PUR50E treatment effectively reversed these changes, restoring glycine and homoserine metabolite levels. These findings indicate that PUR50E may regulate glycine, serine and threonine metabolism to re-establish amino acid balance. Considering that glycine serves as a precursor for GSH synthesis and participates in cytoprotective one-carbon metabolism, the restoration of glycine availability may contribute to reduced oxidative stress and improved hepatocyte survival.

Together, these findings highlight that PUR50E exerts hepatoprotective effects through the modulation of multiple metabolic pathways, including energy metabolism, immune and inflammatory responses, and amino acid homeostasis. By targeting specific metabolites within these pathways, PUR50E not only demonstrates therapeutic efficacy but also uncovers potential metabolic targets that may be exploited for the development of novel strategies against liver fibrosis.

Network pharmacology provides rapid research direction, while metabolomics offers precise experimental validation, avoiding potential bias from single methodologies and enhancing scientific rigor and reliability. This study demonstrates that PUR50E effectively alleviates liver fibrosis through multiple mechanisms. However, our research has limitations that should be addressed in future studies. Our investigation used only a mouse model, which cannot fully replicate human conditions; therefore, our findings need validation in additional animal models and clinical trials. CCl₄-induced fibrosis model primarily reflects toxicant-driven liver injury, the key mechanisms involved in this model are also shared by liver fibrosis arising from other etiologies, including metabolic and cholestatic diseases. Therefore, PUR50E may potentially exert beneficial effects in other fibrosis models. However, direct extrapolation should be approached with caution, and further studies using alternative models, such as diet-induced or bile duct ligation models, are required to validate these effects.

Although the pharmacokinetics of *Pueraria lobata* (Willd.) Ohwi and its components (such as puerarin) have been extensively studied and documented in the literature 16, 43, our current work did not directly assess the pharmacokinetic behavior of PUR50E. Future research could integrate PK/PD models to connect dosage, tissue distribution, and anti-fibrotic efficacy. Additionally, hepatic stellate cells (HSCs)—the primary mediators of liver fibrosis—were not included in this study. Future experiments involving primary HSCs or *in vivo* HSC-specific markers could better elucidate the cellular targets of PUR50E. While metabolomics identified key pathways and metabolites, protein-level validation of the corresponding enzymes is needed to clarify the causal mechanisms of PUR50E-mediated antifibrotic effects. Addressing these limitations will contribute to a more comprehensive understanding of PUR50E's therapeutic potential and support its clinical application.

## 5. Conclusion

**Figure 9:**
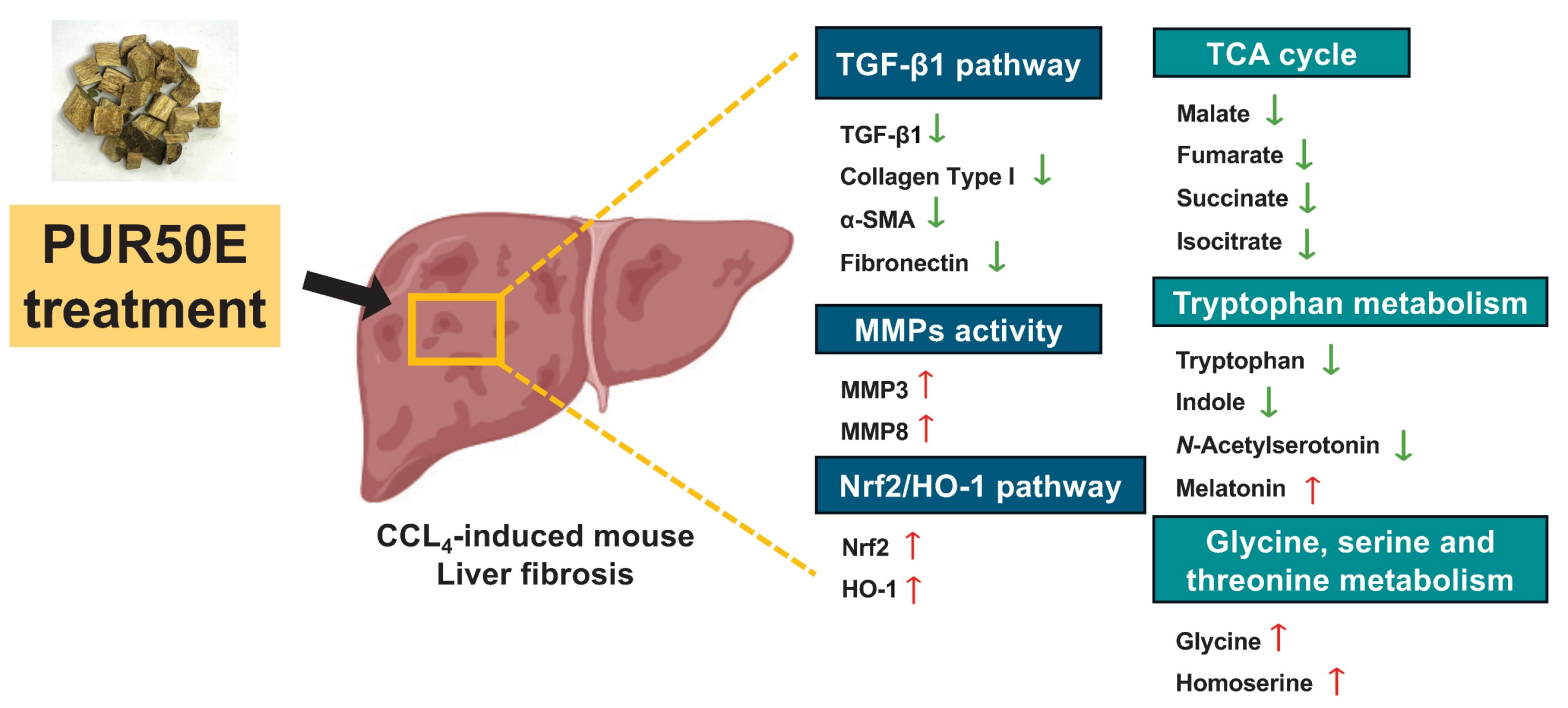
Summary diagram of the underlying mechanisms of the protective effect of PUR50E on CCl₄-induced liver fibrosis.

This study integrated network pharmacology and metabolomics to elucidate the potential protective mechanisms of PUR50E against CCl₄-induced liver fibrosis in mice. PUR50E alleviates liver fibrosis by regulating ECM remodeling, activating the Nrf2/HO-1 antioxidant pathway, and modulating key metabolic pathways including the TCA cycle, tryptophan metabolism, and glycine/serine/threonine metabolism. The identification of critical metabolites—such as malate, fumarate, succinate, isocitrate, tryptophan, indole, *N*-acetylserotonin, melatonin, glycine, and homoserine—not only provides mechanistic insight into disease modulation but also potential biomarkers and therapeutic targets (Fig. 9). These findings provide experimental evidence for the hepatoprotective effects of PUR50E and offer deeper insight into the mechanistic basis of traditional Chinese medicine in liver fibrosis intervention.

## Supplementary Material

Supplementary figures and table.

## Figures and Tables

**Table 1:** The structural information of a 50% ethanolic extract from the *Pueraria lobata* (Willd.) Ohwi in positive-ion mode.

No.	RT (min)	Adduct ion (*m/z*)	Formula	Error (ppm)	MS/MS	Compounds
**1**	5.74	595.1597 [M+H]^+^	C_27_H_30_O_15_	11.1	367 (100), 415 (98), 313 (78), 595 (76)	3'-hydroxypuerarin-4′- O-glucoside
**2**	5.81	579.1678 [M+H]^+^	C_27_H_30_O_14_	6.19	399 (100), 297 (86)	Puerarin-4'-O-glucoside or 6''-O-α-D- glucopyranosylpuerarin or puerarin-7-O- glucoside
**3**	8.82	433.1091 [M+H]^+^	C_21_H_20_O_10_	10.1	283 (100)	3'-hydroxypuerarin
**4**	8.84	565.1493 [M+H]^+^	C_26_H_28_O_14_	11.4	313 (100), 433 (69), 415 (46), 367 (35), 283 (35)	3'-hydroxy-6''-O- xylosylpuerarin or 3'- hydroxypuerarin-6''-O- apioside or its isomer
**5**	9.72	417.1136 [M+H]^+^	C_21_H_20_O_9_	12.0	297 (100), 267 (95), 321 (25)	Puerarin
**6**	10.25	547.1466 [M-H]^-^	C_26_H_28_O_13_	2.61	295 (100), 267 (73), 547 (49)	Daidzein 8-C-apiosyl (1→6) glucoside or its isomers
**7**	10.56	447.1245 [M+H]^+^	C_22_H_22_O_10_	10.3	327 (100), 297 (67), 411 (28), 351 (27), 381 (26)	3'-methoxypuerarin
**8**	11.03	549.1546 [M+H]^+^	C_26_H_28_O_13_	11.3	297 (100), 417 (84), 399 (45), 267 (38)	Puerarin-6″-O-xyloside
**9**	11.15	549.1578 [M+H]^+^	C_26_H_28_O_13_	5.50	297 (100), 417 (85), 351 (63), 399 (39), 267 (33)	Mirificin
**10**	12.51	417.1121	C_21_H_20_O_9_	15.5	255 (100), 199 (8), 181 (8)	Daidzin
**11**	12.88	563.1404 [M-H]^-^	C_26_H_28_O_14_	0.56	311 (100), 283 (38), 563 (28)	Genistein 6-C-[α-D- apiofuranosyl-(1→6)]- β-D-glucopyranoside or 3'-hydroxy-6''-O- xylosylpuerarin or 3'- hydroxymirificin or its isomer
**12**	13.41	447.1236 [M+H]^+^	C_22_H_22_O_10_	12.4	285 (100), 270 (27), 253 (19), 225 (8), 137 (7)	3′-methoxy daidzein-7-O-glucoside
**13**	14.72	447.1246 [M+H]^+^	C_22_H_22_O_10_	10.1	285 (100), 270 (31), 225 (12), 327 (13)	Calycosin-7-O-β-D- glucoside
**14**	14.87	415.1031 [M-H]^-^	C_21_H_20_O_9_	0.46	267 (100), 295 (30), 277 (11)	Daidzein-8-C-α-D- glucoside or daidzein 4'- O-glucoside
**15**	16.37	433.1076 [M+H]^+^	C_21_H_20_O_10_	13.6	271 (100), 215 (8), 153 (6)	Genistein-4′-O-glucoside
**16**	16.61	563.1725 [M+H]^+^	C_27_H_30_O_13_	7.05	311 (100), 365 (64), 395 (47)	genistein 6-C-[α-D- apiofuranosyl-(1→6)]- β-D-glucopyranoside or 3'-hydroxy-6''-O- xylosylpuerarin or 3'- hydroxymirificin or its isomer
**17**	16.85	533.1263 [M+H]^+^	C_25_H_24_O_13_	6.04	285 (100), 270 (70)	5-hydroxy genistein-4′-O-(6″-malonyl)glucoside
**18**	17.72	503.1133 [M+H]^+^	C_24_H_22_O_12_	11.2	255.0623 (100), 199 (6), 227 (3)	Daidzein-4′-O-(6″-malonyl)glucoside
**19**	20.19	459.1255 [M+H]^+^	C_23_H_22_O_10_	7.90	255 (100), 227 (12), 199 (3)	6”-O-acetyldaidzin
**20**	20.91	431.1288 [M+H]^+^	C_22_H_22_O_9_	12.6	269 (100), 254 (9), 237 (8), 226 (7)	Ononin
**21**	22.12	255.0622 [M+H]^+^	C_15_H_10_O_4_	13.9	152 (100), 181 (74), 137 (48), 128 (40)	Daidzein
**22**	22.46	431.1290 [M+H]^+^	C_22_H_22_O_9_	12.1	269 (100), 226 (36)	Isoonoin
**23**	23.12	285.0733 [M+H]^+^	C_16_H_12_O_5_	10.5	213 (100), 137 (83), 197 (45), 269 (29)	Calycosin
**24**	25.44	517.1284 [M+H]^+^	C_25_H_24_O_12_	12.0	269 (100), 237, 213	Formononetin-7-O-(6″-malonyl) glucoside

## Data Availability

The datasets generated during and/or analysed during the current study are available from the corresponding author on reasonable request.

## Abbreviations

α-SMA: α-smooth muscle actin
BP: biological process
CC: cellular composition
Col-I: collagen type I
DDA: data-dependent acquisition
ECM: extracellular matrix
GO: gene ontology
H&E: hematoxylin and eosin
HCC: hepatocellular carcinoma
HSCs: hepatic stellate cells
KEGG: Kyoto Encyclopedia of Genes and Genomes
MF: molecular function
MMPs: matrix metalloproteinases
Nrf2: nuclear factor erythroid 2-related factor 2
OMIM: Online Mendelian Inheritance in Man
OPLS-DA: orthogonal projections to latent structures discriminant analysis
PCA: principal component analysis
PUR50E: *Pueraria lobata* (Willd.) Ohwi 50% ethanol extract
ROS: reactive oxygen species
Sily: silymarin
TCA cycle: citrate cycle
TCM: Traditional Chinese Medicine
TGF-β1: transforming growth factor-β1
TIMPs: tissue inhibitors of metalloproteinases
VIP: variable importance in projection
