# Supplementary material for: Metabolomics Reveals the Anti-hepatic Fibrosis Mechanisms of Pueraria lobata (Willd.) Ohwi Extract and Potential Metabolites Alterations
Source: Int J Med Sci. 2026 Mar 17;23(4):1491–508. doi: 10.7150/ijms.129139 (PMC13048894; doi:10.7150/ijms.129139)
Supplement: Supplementary file 1 — Supplementary figures and table. [file ijmsv23p1491s1.pdf]

Supplementary Materials

Table 1 Primer sequences for qPCR

Gene	Primer sequence (5' to 3')
**Mouse Collagen Type1**	(F)-ATCTCCTGGTGCTGATGGAC<br>(R)-ACCTTGTTTGCCAGGTTCAC
**Mouse α-SMA**	(F)-GTGTTGCCCCTGAAGAGCAT<br>(R)-GCTGGGACATTGAAAGTCTCA
**Mouse Fibronectin**	(F)-CGAGGTGACAGAGACCACAA<br>(R)-CTGGAGTCAAGCCAGACACA
**Mouse TGFβ1**	(F)-TGATACGCCTGAGTGGCTGTCT<br>(R)-CACAAGAGCAGTGAGCGCTGAA
**Mouse MMP3**	(F)-TTGTTCTTTGATGCAGTCAGC<br>(R)-TGATTTGCGCCAAAAGTGC
**Mouse MMP13**	(F)-GATGACCTGTCTGAGGAAGACC<br>(R)-GCATTTCTCGGAGCCTGTCAAC
**Mouse Nrf2**	(F)-CTATGCAGGCTGTGGCAAAACC<br>(R)-TTGCGGTAGTGCCTGGTCAGTT
**Mouse HO-1**	(F)-CTGAACTCCTGGACGGGACTA<br>(R)-CGGTGGGTCTCCGTAAATGG
**Mouse GAPDH**	(F)-TGACCTCAACTACATGGTCTACA<br>(R)- CTTCCCATTCTCGGCCTTG

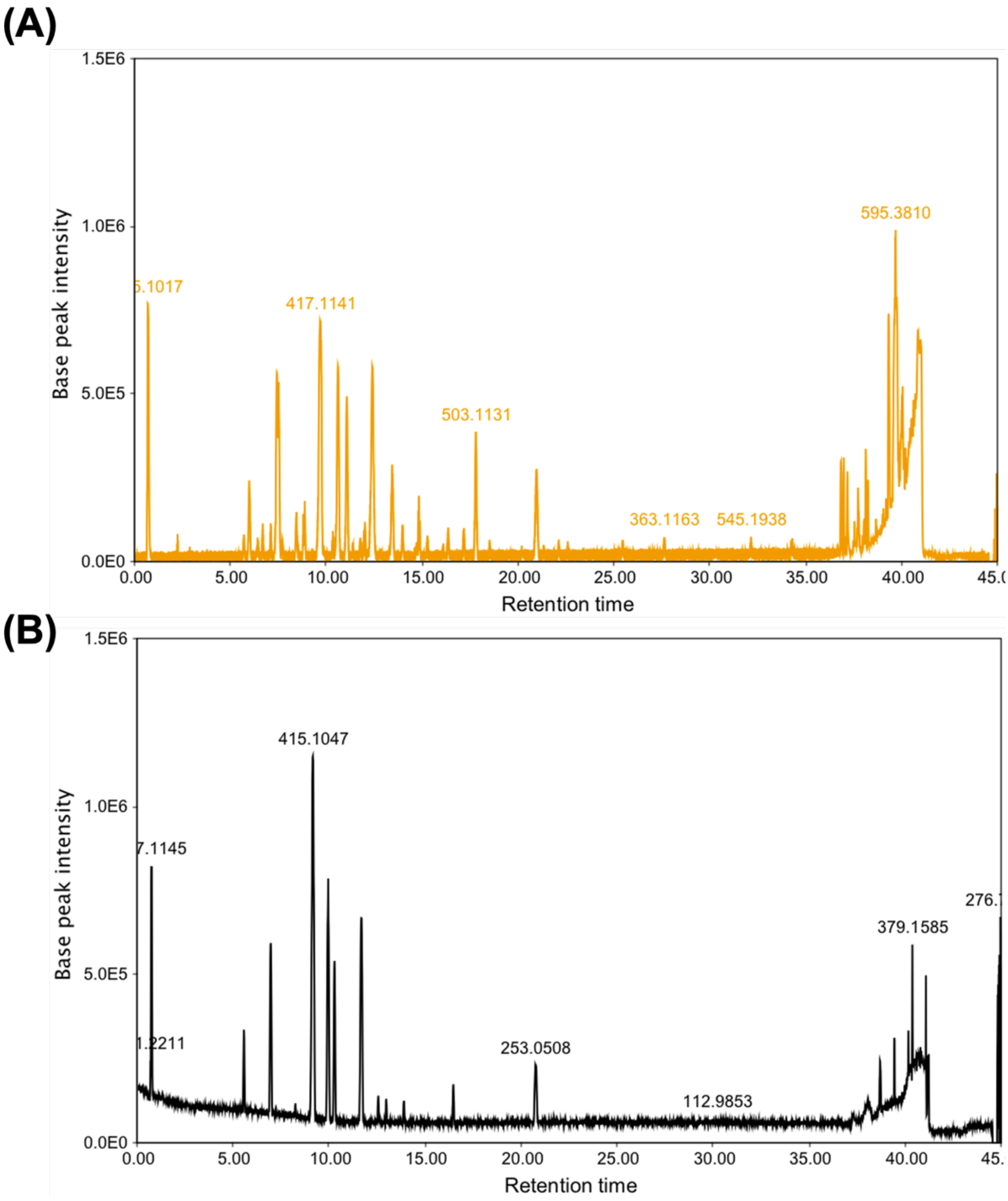

Figure S1. HPLC-QTOF-MS/MS of ESI+ (A) and ESI- (B).

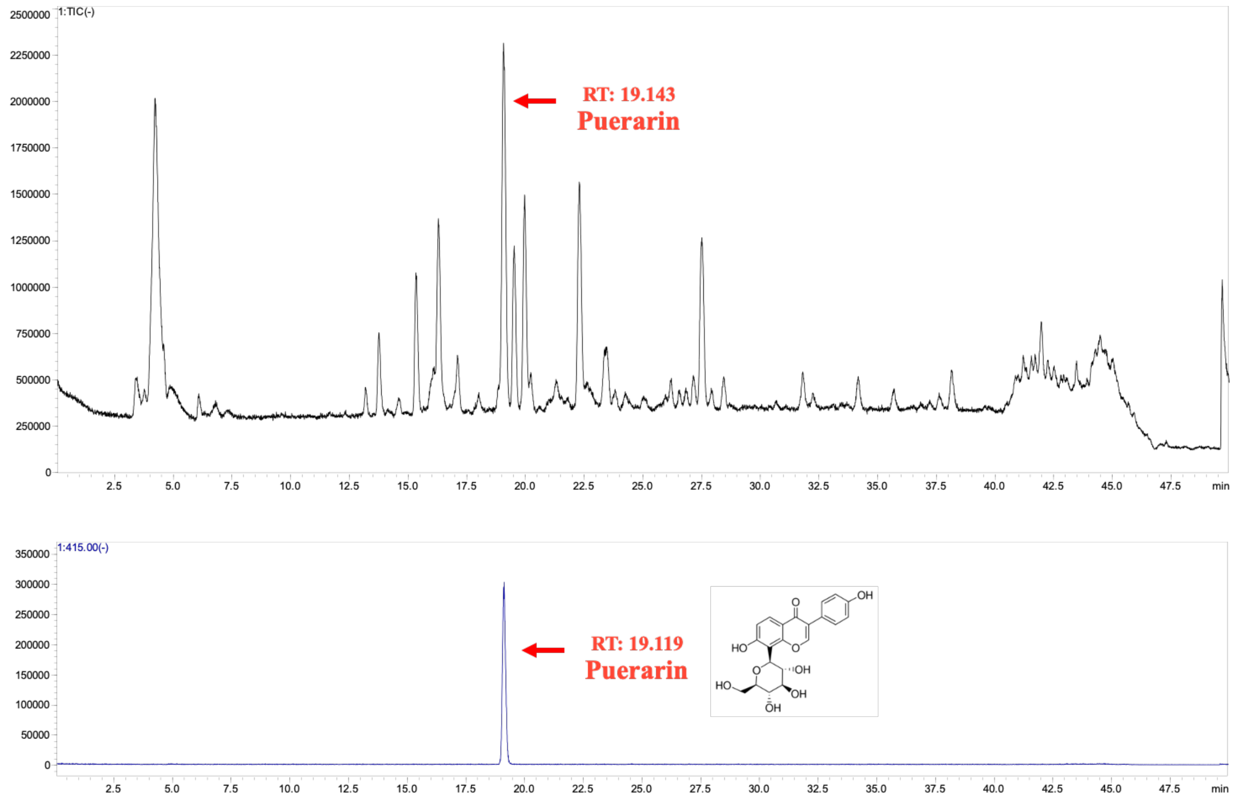

Figure S2. Total ion chromatogram (TIC) of PUR50E acquired in negative ion mode. The peak observed at a retention time of 19.1 min corresponds to the standard compound puerarin.

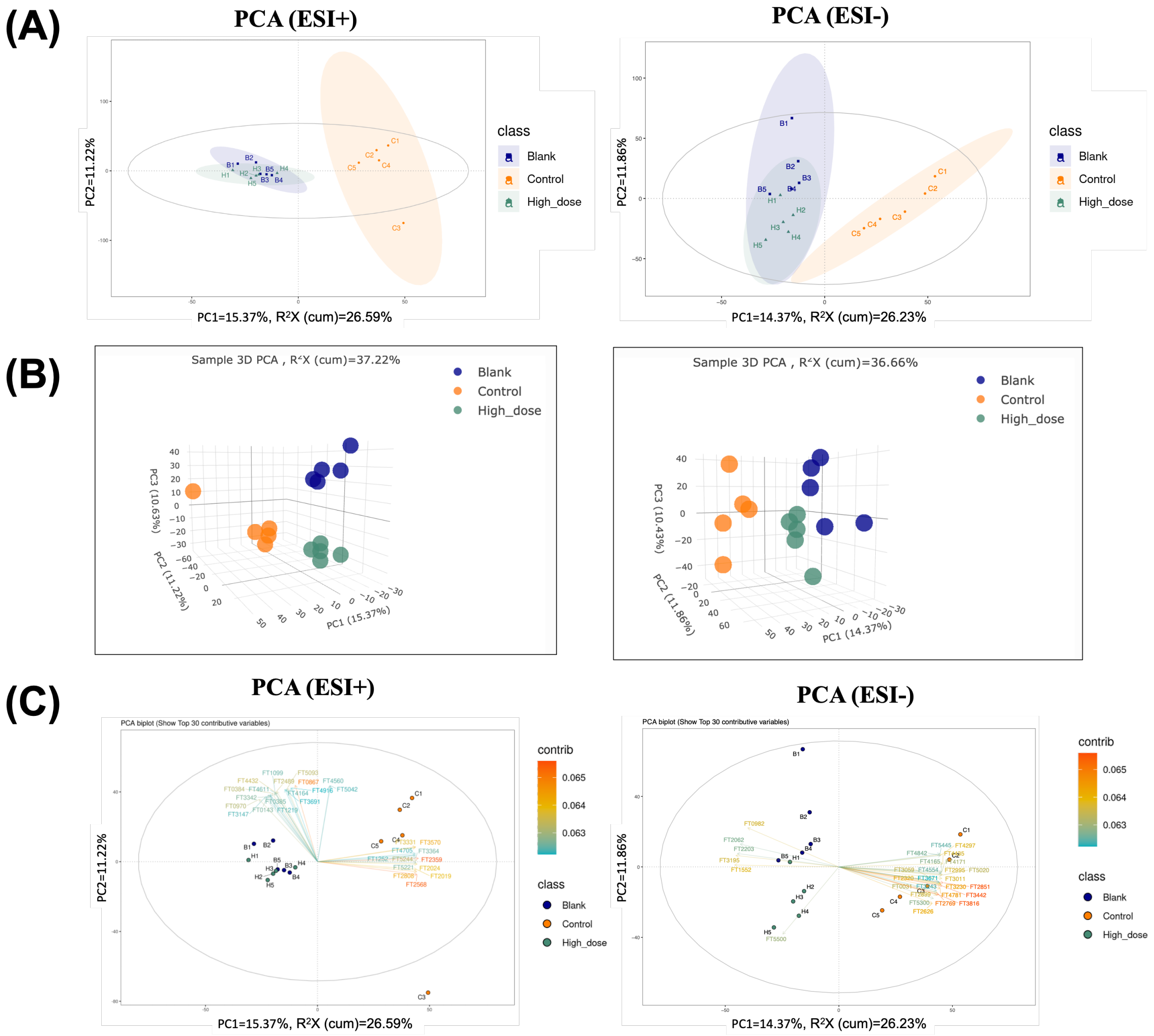

Figure S3. Plots of serum PCA scores between positive and negative ion normal Blank group, Control group (CCl4-induced) and High_does (PUR-H) group mouse. (A) 2D score plots in both ESI+ and ESI− modes, where each point represents a sample, and samples in the same group are represented by the same color. (B) 3D PCA plots in ESI+ and ESI− modes. (C) Biplot in ESI+ and in ESI− mode. Each arrow represents a feature: its direction indicates contribution, its length reflects magnitude (longer = higher contribution), and its angle with the axes shows correlation with the principal components. Arrow colors indicate contribution levels (%) to PC1 and PC2, with orange-red showing higher contributions and blue indicating lower.
